# Supplementary material for: Long-read transcriptome analysis using IsoRanker for identifying pathogenic variants in Mendelian conditions
Source: medRxiv. 2025 Nov 13:2025.11.07.25339764. Preprint. [Version 1] doi: 10.1101/2025.11.07.25339764 (PMC12642736; doi:10.1101/2025.11.07.25339764)
Supplement: 1 [file NIHPP2025.11.07.25339764V1-supplement-1.pdf]

medRxiv preprint doi: https://doi.org/10.1101/2025.11.07.25339764; this version posted November 13, 2025. The copyright holder for this preprint (which was not certified by peer review) is the author/funder, who has granted medRxiv a license to display the preprint in perpetuity.

It is made available under a CC-BY 4.0 International license .

Supplementary Figures

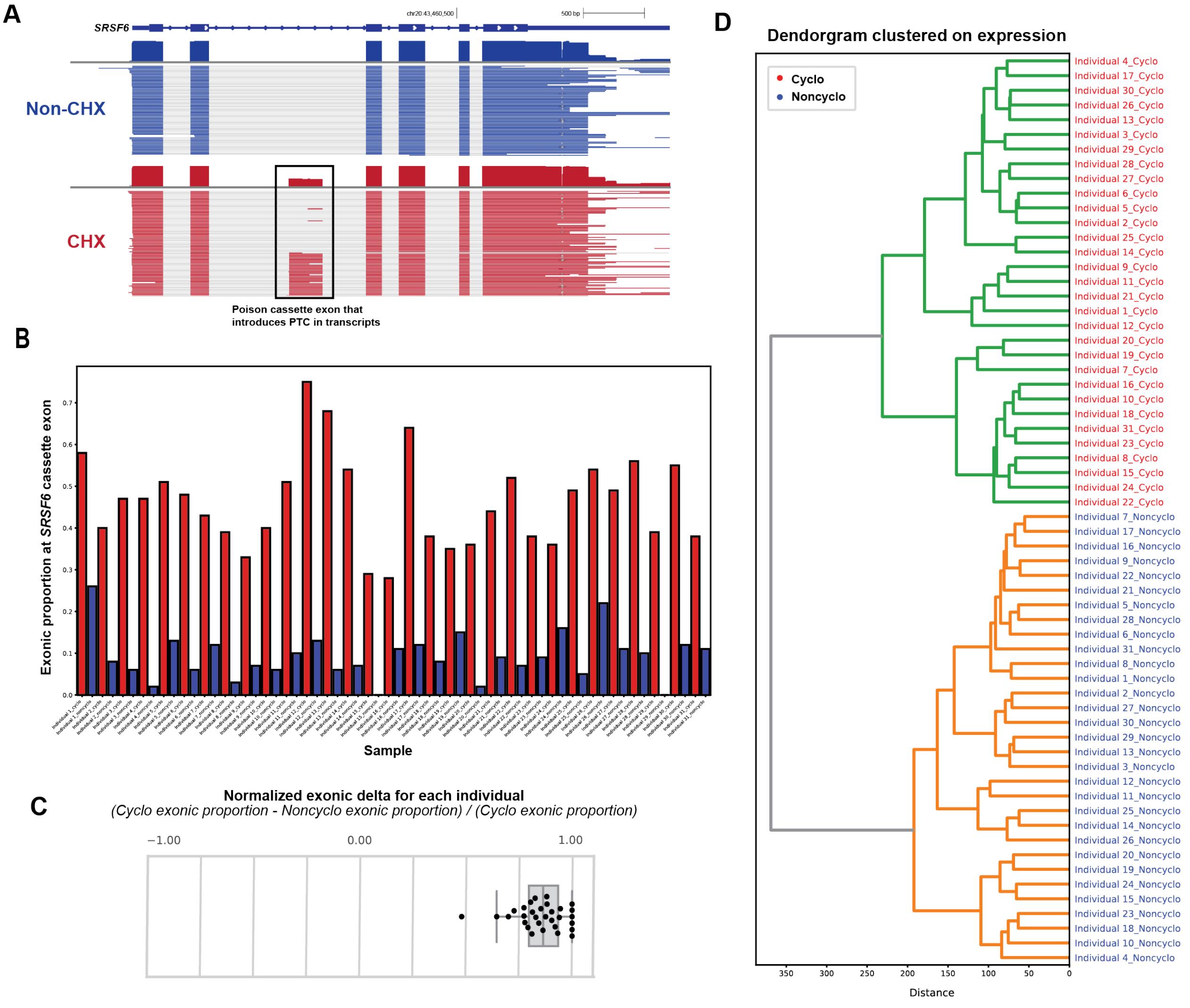

Supplementary Figure 1. Validating effects of cycloheximide:
(**A**) *SRSF6* harbors a poison cassette exon that introduces a premature termination codon (PTC). When this exon is included in the transcript, the transcript is degraded via NMD during steady-state. Thus, detection of the cassette exon when a sample is treated with CHX is useful for validating the effects of CHX on inhibiting NMD.
(**B**) For each individual, the proportion of *SRSF6* reads with inclusion of the cassette exon was calculated and plotted for the CHX-treated (red) sample and the untreated sample (blue). In each individual, the CHX-treated sample had much greater proportion of reads with the NMD-inducing cassette exon. This validated the effect of CHX on inhibiting NMD for each individual.
(**C**) The normalized exonic delta at the position of the *SRSF6* cassette exon for each individual was calculated and plotted.
(**D**) Gene-level expression matrix was used for hierarchical clustering and generation of a dendrogram.

medRxiv preprint doi: https://doi.org/10.1101/2025.11.07.25339764; this version posted November 13, 2025. The copyright holder for this preprint (which was not certified by peer review) is the author/funder, who has granted medRxiv a license to display the preprint in perpetuity.

It is made available under a CC-BY 4.0 International license .

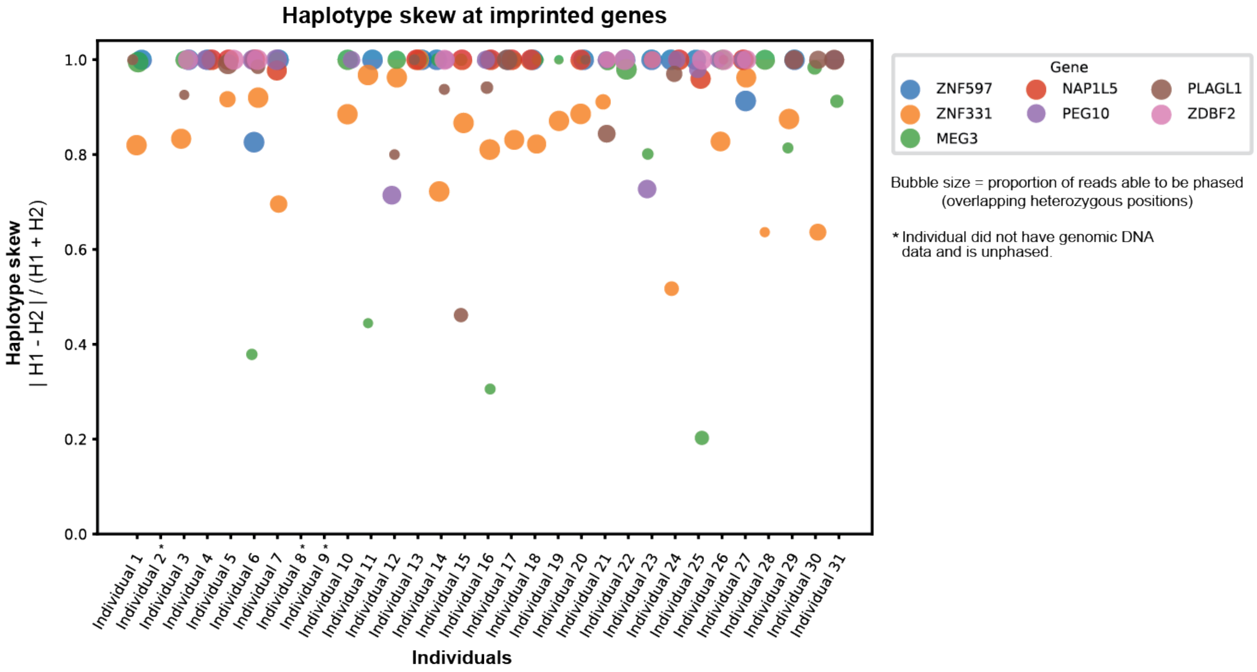

Supplementary Figure 2. Validating ability to phase transcript data: Seven genes known to be imprinted and expressed in fibroblasts were selected to be used to evaluate our ability to appropriately phase transcript data. The majority of expression from these genes showed strong haplotype skewing, indicating successful phasing of transcripts.

medRxiv preprint doi: https://doi.org/10.1101/2025.11.07.25339764; this version posted November 13, 2025. The copyright holder for this preprint (which was not certified by peer review) is the author/funder, who has granted medRxiv a license to display the preprint in perpetuity.

It is made available under a CC-BY 4.0 International license .

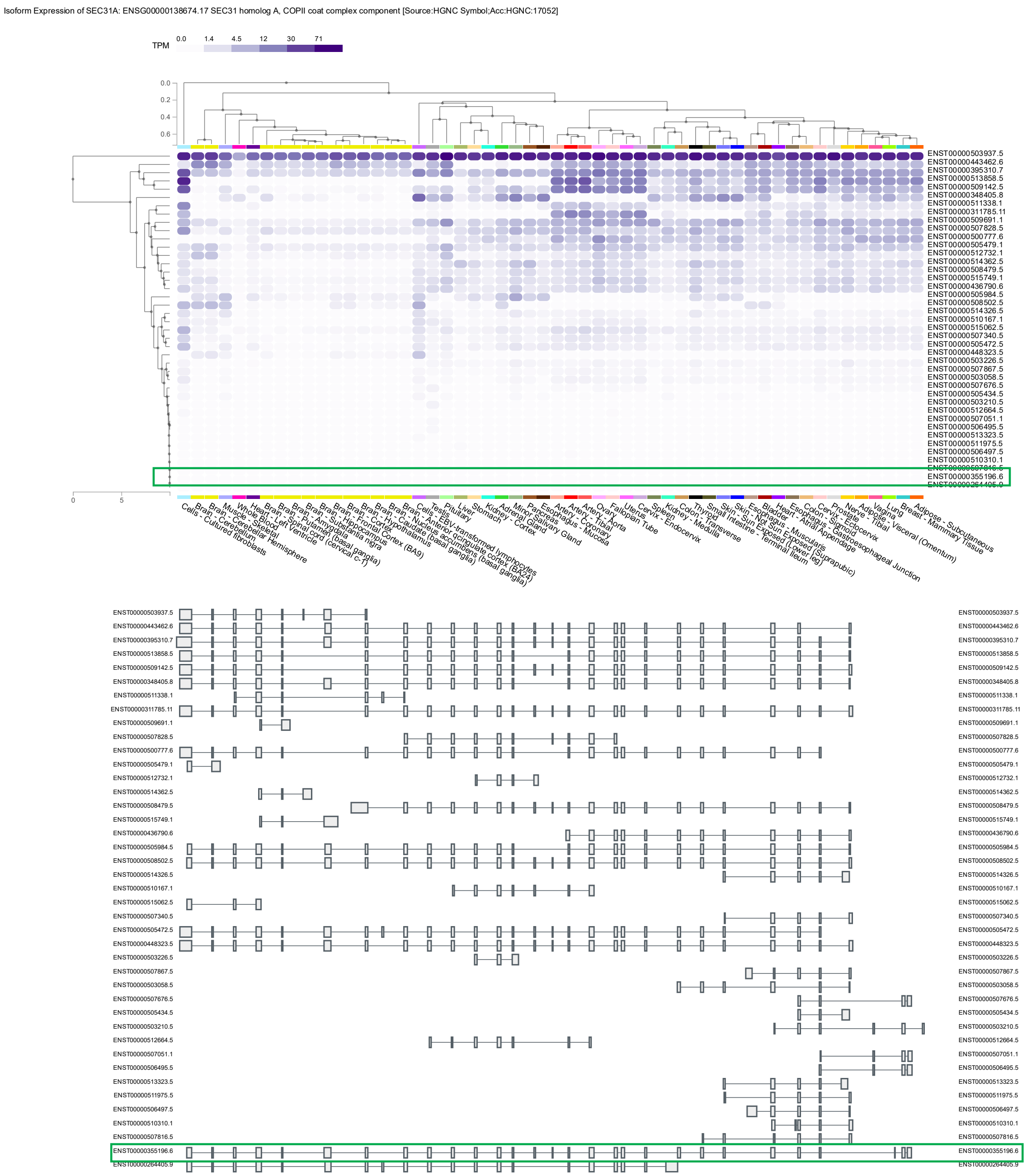

Supplementary Figure 3. *SEC31A* GTEx isoform expression: The *SEC31A* isoform (ENST00000355196.6) that overlaps the splice donor variant in Individual 5 is a minimally expressed isoform in all tissues.

medRxiv preprint doi: https://doi.org/10.1101/2025.11.07.25339764; this version posted November 13, 2025. The copyright holder for this preprint (which was not certified by peer review) is the author/funder, who has granted medRxiv a license to display the preprint in perpetuity.

It is made available under a CC-BY 4.0 International license .

Supplementary Tables

Variant type	Individual	Variant (hg38)	Zygosity	Impact	cDNA	Gene	Noncyclo expression TPM	Cyclo expression TPM
Candidate	Individual 1	chr7:107258479C>T	het	splice donor variant	ENST00000468350.1:n.141+1G>A	*COG5*	61.13	48.53
Candidate	Individual 5	chr4:82900333C>A	het	splice donor variant	ENST00000355196.6:c.-174+1G>T	*SEC31A*	367.44	349.79
Candidate	Individual 9	chr2:190985665A>G	het	splice region variant	ENST00000361099.7:c.1222-5T>C	*STAT1*	824.86	1910.01
Candidate	Individual 11	chr12:104889178C>T	het	splice acceptor variant	ENST00000258538.3:c.736-1G>A	*SLC41A2*	11.04	22.25
Candidate	Individual 17	Deletion chr5:123388032-123388069	hom	intronic deletion	N/A	*CEP120*	10.78	5.76
Candidate	Individual 18	chr3:9697867G>C	het	splice donor variant	ENST00000296003.4:c.1769+1G>C	*MTMR14*	66.66	62.79
Candidate	Individual 20	Deletion chr22:49914596-49924246	het	upstream deletion	N/A	*ALG12*	29.17	17.73
Candidate	Individual 21	chr11:71435435G>A	het	splice region variant	ENST00000533800.5:c.611+7C>T	*DHCR7*	62.50	45.67
Candidate	Individual 21	chr9:127682406C>T	het	splice region variant	ENST00000373302.7:c.1548C>T	*STXBP1*	42.45	59.94
Candidate	Individual 22	chr6:85536924CCTGAA>C	het	splice acceptor variant	ENST00000314673.8:c.1476-5_1476-1del	*SNX14*	76.23	37.56
Known transcript-altering	Individual 1	Duplication chr6:142150876-142179070	het	28kb duplication	N/A	*VTA1*	91.24	106.42
Known transcript-altering	Individual 2	chr9:128693813G>C	het	splice donor 5th base variant	ENST00000322030.13:c.663+5G>C	*SET*	176.91	125.91
Known transcript-altering	Individual 3	chr1:11998739T>G	hom	intronic splice branchpoint variant	ENST00000235329.10:c.600-31T>G	*MFN2*	18.18	19.53
New diagnosis	Individual 6	chr5:140673926T>A	het	3 prime UTR variant	ENST00000504156.7:c.*331A>T	*HARS1*	15.62	28.66
New diagnosis	Individual 6	chr5:140676984C>A	het	splice donor 5th base variant	ENST00000504156.7:c.951+5G>T	*HARS1*	15.62	28.66

Supplementary Table 1. Details of genetic variants: The table summarizes the specific variants highlighted in the manuscript, including their type, genomic coordinates (hg38), zygosity, predicted functional impact, and affected isoform (cDNA) and gene. For each individual carrying the variant, gene expression levels (in transcripts per million, TPM) are shown under noncycloheximide (Noncyclo) and cycloheximide (Cyclo) treatment conditions.

medRxiv preprint doi: https://doi.org/10.1101/2025.11.07.25339764; this version posted November 13, 2025. The copyright holder for this preprint (which was not certified by peer review) is the author/funder, who has granted medRxiv a license to display the preprint in perpetuity.

It is made available under a CC-BY 4.0 International license .

Individual	Sample	Downsampled Total read cou	Mean transcript leng	Read length N	Unique ger	Unique isofor	Reads in novel isofor	Unique novel isofor	Phased rea	Means isoforms pe	Parental data
Individual 1	noncyclo	6575851	2198.338217	2414	38558	370896	1295529	236377	3240039	9.619171119	yes
Individual 1	cyclo	6512075	2415.057453	2520	38732	429851	1554876	297957	3170703	11.09808427	yes
Individual 2	noncyclo	5562276	2201.363523	2304	34041	322332	1164316	206532	0	9.46893452	
Individual 2	cyclo	5138602	2215.295438	2325	31652	355634	1173342	246957	0	11.2357513	
Individual 3	noncyclo	4951319	1993.575428	2112	38404	313994	984259	188774	1689905	8.176075409	
Individual 3	cyclo	4966286	2152.055394	2240	31444	343934	1115253	235813	1719344	10.93798499	
Individual 4	noncyclo	4969730	1713.44469	1860	31915	244512	980908	146199	2013048	7.661350462	
Individual 4	cyclo	4970710	2027.574945	2117	58461	318668	1066470	190370	2401943	5.450950206	
Individual 5	noncyclo	5339769	2163.114136	2284	72453	349559	1041692	191771	2215967	4.82463114	
Individual 5	cyclo	4388409	2278.912593	2343	42610	341325	969037	223435	1764150	8.010443558	
Individual 6	noncyclo	5443256	2191.590621	2303	44742	362042	1129051	221341	2404408	8.091770596	yes
Individual 6	cyclo	5582614	2265.861489	2335	33791	370208	1247085	254422	2585879	10.95581664	yes
Individual 7	noncyclo	6052316	2070.152623	2145	38972	357030	1256867	223026	2843258	9.161192651	yes
Individual 7	cyclo	4961840	1765.947058	1877	57592	382500	1127822	242102	2253713	6.641547437	yes
Individual 8	noncyclo	6091477	1967.804938	2091	50932	345095	1212184	202130	0	6.775602764	
Individual 8	cyclo	6547627	1944.90596	2115	39169	389940	1373861	257413	0	9.955321811	
Individual 9	noncyclo	5947763	2321.955581	2467	33692	363250	1292628	239214	0	10.78149116	
Individual 9	cyclo	3926173	3104.232909	3619	28317	362940	1039933	261932	0	12.8170357	
Individual 10	noncyclo	6458978	1745.927358	1907	34075	310457	1213185	193386	2606903	9.110990462	yes
Individual 10	cyclo	5845121	2161.723603	2366	76237	454716	1334940	277671	2485720	5.964505424	yes
Individual 11	noncyclo	4980351	2704.032495	3128	33039	332433	1138656	216049	2330093	10.06183601	
Individual 11	cyclo	5527613	2532.712241	2634	34270	419415	1331292	300623	2494712	12.23854683	
Individual 12	cyclo	6680205	2149.478551	2332	45816	497214	1608883	346216	2867310	10.85240964	yes
Individual 12	noncyclo	4322437	2594.465984	3124	44400	357397	981915	218193	1775446	8.049481982	yes
Individual 13	cyclo	4961078	2155.9225	2331	31977	346531	1131136	237785	2079376	10.83688276	yes
Individual 13	noncyclo	4963610	2007.226684	2131	29493	281797	962673	180306	2075004	9.554707897	yes
Individual 14	cyclo	6620931	2221.54652	2511	36003	433920	1491465	301228	2976771	12.05232897	
Individual 14	noncyclo	6277609	2290.630094	2637	34624	366952	1358331	236713	2825933	10.59819778	
Individual 15	noncyclo	4961782	1722.209577	1844	72867	317526	932439	165766	1987513	4.357610441	yes
Individual 15	cyclo	4969164	1829.447887	2009	35516	302466	984300	193593	2021189	8.516330668	yes
Individual 16	noncyclo	6675588	2051.785567	2134	47758	437358	1433298	277970	2606253	9.157795553	yes
Individual 16	cyclo	4962142	1947.431189	2074	33473	331450	1108403	217979	2011335	9.902010576	yes
Individual 17	noncyclo	5657151	1978.218565	2119	41812	366179	1127959	226643	2122001	8.757748972	
Individual 17	cyclo	5206297	2149.862863	2218	43563	354805	1083293	226696	1987473	8.144641095	
Individual 18	noncyclo	4965226	1705.853747	1862	31081	253713	950131	152180	1650068	8.162961295	yes
Individual 18	cyclo	4969073	2141.848636	2246	48306	348879	1105024	220004	1897970	7.222270525	yes
Individual 19	noncyclo	4956637	1751.084243	1909	70972	326506	949028	173071	1967823	4.600490334	yes
Individual 19	cyclo	4957944	1624.648171	1806	36685	304997	968170	196467	1997496	8.313943028	yes
Individual 20	noncyclo	6650800	1620.474638	1805	34045	297041	1204252	183981	2466649	8.724952269	
Individual 20	cyclo	4962538	1671.007452	1844	31805	291948	985273	189336	1871198	9.179311429	
Individual 21	noncyclo	5936197	1888.857039	1991	43850	349728	1237468	211409	2188888	7.975553022	
Individual 21	cyclo	4904563	2661.280414	2920	37339	465257	1395242	329369	2067695	12.46034977	
Individual 22	noncyclo	5667551	2056.214125	2142	37130	346656	1191416	222973	2299976	9.336277942	
Individual 22	cyclo	5883791	1777.999173	1910	78454	487834	1460925	298207	2294291	6.218089581	
Individual 23	noncyclo	6256116	1733.129761	1897	54951	337436	1274993	197478	2210204	6.140670779	
Individual 23	cyclo	6287232	2041.805827	2310	41838	455310	1428561	307132	2400639	10.88269038	
Individual 24	noncyclo	4957855	1778.545215	1920	42449	315544	990151	186610	1949310	7.433484888	
Individual 24	cyclo	4953616	1695.035326	1884	44886	323197	1046322	200108	1821873	7.20039656	
Individual 25	cyclo	5701034	2303.624891	2682	41880	461811	1358640	315130	2776975	11.02700573	
Individual 25	noncyclo	5646163	2255.435189	2697	43280	375687	1171341	231066	2688015	8.680383549	
Individual 26	cyclo	6655248	2156.446563	2349	43276	423768	1426505	282601	2795962	9.792217395	yes
Individual 26	noncyclo	4374748	2342.876987	2580	43513	338246	927630	201971	1899049	7.773447016	yes
Individual 27	cyclo	5299251	2291.660344	2500	33079	359124	1156074	241451	2789264	10.85655552	
Individual 27	noncyclo	6227031	2134.560341	2336	36474	335973	1225234	209805	3139154	9.211301201	
Individual 28	cyclo	3337545	2260.598629	2395	30002	279294	747418	182302	1497675	9.309179388	yes
Individual 28	noncyclo	4746131	2067.549258	2177	66157	336273	943454	183367	2037324	5.082954185	yes
Individual 29	cyclo	5115653	2285.644279	2341	28556	338656	1168968	237572	2514212	11.85936406	yes
Individual 29	noncyclo	5604432	2046.860063	2118	30261	305767	1136160	200591	2585109	10.1043257	yes
Individual 30	cyclo	4958807	2070.766282	2165	34204	334835	1074690	219687	2880851	9.789352123	
Individual 30	noncyclo	4958407	2246.846191	2420	33190	305952	1008690	189989	2914082	9.218198252	
Individual 31	noncyclo	5090489	1937.911692	2046	38248	332996	1060214	203799	1924104	8.706233006	
Individual 31	cyclo	5678026	1860.679669	2013	38942	392518	1249976	268806	2090846	10.07955421	
All		336700254	2083.87834	2275	525843	6657006	72089231	5149234	129172091		

Supplementary Table 2. Summary of quality control metrics for long-read transcriptome sequencing data: The table lists, for each individual and sample type, the total number of downsampled reads, mean transcript length, read length N50, number of unique genes and isoforms detected, proportion of reads assigned to novel isoforms, number of unique novel isoforms, number of phased reads, mean isoforms per gene, and availability of corresponding parental data.

medRxiv preprint doi: https://doi.org/10.1101/2025.11.07.25339764; this version posted November 13, 2025. The copyright holder for this preprint (which was not certified by peer review) is the author/funder, who has granted medRxiv a license to display the preprint in perpetuity.

It is made available under a CC-BY 4.0 International license .

EVIDENCE FOR PATHOGENICITY:				
RULE	STRENGTH	BRIEF DESCRIPTION	chr5:140676984C>A (NM_002109.6:c.951+5G>T)	chr5:g.140673926T>A (NM_002109.6:c.*331A>T)
PVS1	Very Strong	Null variant (w/ LOF mechanism)	PVS1	
PS1	Strong	Different nt change (same aa) as a path variant		
PS2	Strong	*De novo* (mat/pat confirmed)		
PS3	Strong	Established functional data shows deleterious		PS3_supporting
PS4	Strong	Case data or case-control studies		
PM1	Moderate	Mutation hotspot/critical functional domain		
PM3	Moderate	*In trans* with path variant (AR)		PM3
PM4	Moderate	In-frame indels, stop-loss variants		
PM5	Moderate	Different aa change at codon is path		
PM6	Moderate	Assumed *de novo* (no mat/pat testing)		
PM2	Supporting	Absent/rare in controls	PM2_supporting	PM2_supporting
PP1	Supporting	Segregation		
PP2	Supporting	Missense in a gene with low rate of variation		
PP3	Supporting	Computational evidence predicts impact		
PP4	Supporting	Disease with single genetic etiology		
PP5	Supporting	Reputable source reports variant as path		
N/A	Any strength	Other evidence not captured by ACMG		
**EVIDENCE FOR BENIGN:**				
**RULE**	**STRENGTH**	**BRIEF DESCRIPTION**	**Applied?**	**Applied?**
BA1	Stand Alone	>5% MAF		
BS1	Strong	MAF greater than disorder frequency		
BS2	Strong	Observed in healthy individual		
BS3	Strong	Established functional data shows no effect		
BS4	Strong	Lack of segregation		
BP1	Supporting	Missense in gene with only truncating mutations		
BP2	Supporting	*In trans* (AD) or *in cis* (all) with path variant		
BP3	Supporting	In-frame indels in repetative region		
BP4	Supporting	Computation evidence predicts no impact		BP4
BP5	Supporting	Case has alternate molecular basis for disease		
BP6	Supporting	Reputable source reports variant as benign		
BP7	Supporting	Synonymous with no splice impact/conservation		
N/A	Any strength	Other evidence not captured by ACMG		
		Classification submitted to ClinVar:	**Likely Pathogenic**	**Uncertain Significance**

Supplementary Table 3. Interpretation of Individual 6 *HARS1* variants using ACMG guidelines.

medRxiv preprint doi: https://doi.org/10.1101/2025.11.07.25339764; this version posted November 13, 2025. The copyright holder for this preprint (which was not certified by peer review) is the author/funder, who has granted medRxiv a license to display the preprint in perpetuity.

It is made available under a CC-BY 4.0 International license .

Individual	External ID
Individual 1	UDN687128
Individual 2	UDN215640
Individual 3	UDN633333
Individual 4	UDN318336
Individual 5	UDN052264
Individual 6	UDN212054
Individual 7	UDN355246
Individual 8	UDN860070
Individual 9	UDN353099
Individual 10	UDN305049
Individual 11	UDN310876
Individual 12	MAN_1199-01
Individual 13	MAN_1877-01
Individual 14	MAN_310-01
Individual 15	UDN121217
Individual 16	UDN374570
Individual 17	UDN827597
Individual 18	UDN966087
Individual 19	UDN204349
Individual 20	UDN359892
Individual 21	UDN262407
Individual 22	UDN924176
Individual 23	UDN666440
Individual 24	UDN178694
Individual 25	MAN_1986-01
Individual 26	MAN_0252-01
Individual 27	MAN_2275-01
Individual 28	MAN_2401-01
Individual 29	MAN_2410-01
Individual 30	MAN_2443-01
Individual 31	UDN010451

Supplementary Table 4. Lookup table for external IDs.
